# Comparison of Cervical Screening at 6‐ and 12‐Weeks Postnatal: A Paired‐Sample Feasibility Study

**DOI:** 10.1111/1471-0528.70234

**Published:** 2026-04-01

**Authors:** Victoria A. Cullimore, Tessa Dean, Kim Chu, Adam Brentnall, Karin Denton, Katherine Hunt, Alexandra Sargent, Laura Johns, Katharine Edey, Sarah L. Coleridge, Jane Borley, Rebecca Newhouse, Holly Baker‐Rand, Lorna McWilliams, Emma J. Davidson, Jo Morrison

**Affiliations:** ^1^ Department of Gynaecological Oncology, GRACE Centre Musgrove Park Hospital, Somerset NHS Foundation Trust Taunton Somerset UK; ^2^ Faculty of Health and Life Sciences University of Exeter Exeter Devon UK; ^3^ Wolfson Institute of Population Health, Queen Mary University of London, Charterhouse Square London UK; ^4^ Department of Cytopathology North Bristol Trust Bristol UK; ^5^ Gynae Cytology Department Manchester University NHS FT Manchester UK; ^6^ Department of Obstetrics and Gynaecology Royal Devon and Exeter Exeter UK; ^7^ Department of Gynaecological Oncology Royal Cornwall Hospital Truro UK; ^8^ Department of Gynaecological Oncology University Hospital Southampton NHS Foundation Trust Southampton Hampshire UK; ^9^ Cancer Prevention & Early Detection Research, NIHR Manchester Biomedical Research Centre, Faculty of Biology, Medicine & Health The University of Manchester, St Mary's Hospital Manchester UK; ^10^ Manchester Centre for Health Psychology, Division of Psychology & Mental Health, School of Health Sciences The University of Manchester Manchester UK

**Keywords:** cervical screening, postnatal, self‐sampling

## Abstract

**Objective:**

Feasibility of a paired‐sample study comparing cervical screening and urine self‐sampling at 6‐ and 12‐weeks postnatal.

**Design:**

Paired‐sample feasibility study.

**Setting:**

Acute hospital.

**Population or Sample:**

Females within 6 weeks of childbirth.

**Methods:**

Paired‐sample study comparing cervical screening and high‐risk human papillomavirus (HPV) testing from clinician‐taken samples and urine self‐samples at 6‐ and 12–weeks postnatal.

**Main Outcome Measures:**

Uptake rates, patient‐reported outcomes, adverse events, HPV detection, relative HPV sensitivity and specificity.

**Results:**

Of 245 potential participants, 115 (47%) consented, 102 (89%) and 96 (83%) attended their 6‐ and 12‐week screening visits, respectively. Median pain scores for cervical screening did not differ (6‐weeks = 1; 12‐weeks = 1; *p* = 0.76). Most would be happy for future screening at 6‐weeks postnatal, 97/102 (95%) and 85/95 (89%) when asked at 6‐ and 12‐week study visits, respectively. Agreement rate of clinician‐taken HPV tests between 6‐ and 12‐weeks was 94.8% (91/96, 95% confidence interval [CI], 88.4%–97.8%), with no inadequate combination HPV/cytology tests (6 vs. 12‐week: sensitivity 80% [95% CI, 44.4–97.5]; specificity 96.5% [95% CI 90.1–99.3]). Relative to 12‐week clinician‐taken samples, urine self‐sampling may have lower sensitivity for HPV (e.g., 6‐week: sensitivity 60.0% [95% CI 26.2–87.8]; specificity 96.5% [95% CI 90.1–99.3]; NPV 95.4% [95% CI 88.6–98.7]).

**Conclusions:**

A paired‐sample study of cervical screening at 6‐ and 12‐weeks is feasible. Inadequacy rates of cervical screening were low, with high agreement. Urine self‐sampling may be less sensitive in this population.

## 1 Introduction

Cervical cancer is highly preventable, with high screening uptake central to the World Health Organisation's (WHO) and the United Kingdom's (UK) National Health Service's (NHS) cervical cancer elimination strategies [1, 2, 3, 4]. Cervical screening rates in England have fallen to the lowest levels since the NHS Cervical Screening Programme (CSP) was introduced in 1988, with only 25% of eligible women screened in some areas, leading to increased risk of cervical cancer [5, 6, 7, 8]. Those less likely to be screened include women with children under 5 years, and those with intersectional disadvantage [9]. In the most recent NHS CSP report, screening uptake was notably lower in women aged under 50, suggesting they face specific barriers or influences that affect their decision to participate [8].

Cervical cancer affects women of reproductive age, with peak incidence in those aged 30–34 years in the UK [10], coinciding with the average age of childbirth (30.9 years) [11]. According to a quality improvement study in Somerset, half of the local maternity population were overdue for cervical screening following childbirth, and the majority had still not attended by 6 months postnatal [12]. In order to address this problem, views of new mothers/birth parents and primary care (General Practitioner [GP]) providers were canvassed for ideas to improve uptake [12]. Postnatal cervical screening alongside the GP 6‐week postnatal check‐up was suggested, to reduce practical barriers, and the option of self‐sampling was welcomed when introduced as part of discussions. National Institute for Health and Care Excellence (NICE) guidelines recommend a GP postnatal check for mothers and babies, attended by 78% of eligible people, which ideally provides opportunities for health promotion [13, 14, 15].

‘Not getting around to’ having a test is an independent barrier to cervical screening, regardless of screening status [16, 17], and the most common reason for younger women being under‐screened [18]. Mixed methods research (quantitative survey and follow‐up semi‐structured interviews) identified that this population experiences misinformation, experiences difficulty making appointments, has competing priorities and perceives postnatal infant care to be prioritised over maternal health [19, 20].

Guidelines, both international and in the UK, advise delaying routine cervical screening until 12‐weeks postnatal, if routine cervical screening is due in pregnancy [21, 22], largely based on a cohort study evaluating Papanicolaou smears at 4‐, 6‐ and 8‐weeks postnatal [23]. This evidence is inadequate because it pre‐dates the introduction of high‐risk human papillomavirus (HPV) testing and liquid‐based cytology (LBC). Studies using LBC have shown no difference in inadequate sample rates at 6‐weeks postnatal compared with a non‐pregnant population (not HPV tested) [24], suggesting that current recommendations may be outdated in the era of HPV‐testing and LBC.

Previous studies investigated acceptability of cervical screening at 6‐weeks postnatal [19, 20]. These data suggest many women would be more likely to have cervical screening, if offered at the GP postnatal check‐up, and more if this specifically involved urine self‐sampling [19].

### 1.1 Objective

We aimed to assess the acceptability and feasibility of a paired study design to evaluate conventional cervical screening and self‐sampling at 6 weeks versus 12 weeks postnatal, in line with the Medical Research Council guidance for evaluating complex interventions [25], and a framework for feasibility study design prior to randomised control trials [26]. The primary objective of this study was to evaluate the feasibility of a paired‐sample study design for a future larger scale trial investigating the acceptability of cervical screening at 6 weeks postnatal and willingness to have repeat screening at 12 weeks postnatal. Secondary objectives were to: evaluate the acceptability of clinician‐taken cervical samples and self‐collected urine samples; assess the quality of cervical samples at 6 weeks postnatal through inadequacy rates; and to determine the agreement in hrHPV status at 6 weeks and 12 weeks postnatal between clinician‐taken cervical samples and self‐collected urine samples.

## 2 Methods

A feasibility study [25], with a target of 100 participants, was designed and conducted [25, 27]. Primary outcomes for feasibility were recruitment rates and completion of screening visits. Secondary outcomes were participant reported outcomes, adequacy rates of cervical screening and agreement in HPV results of 6‐week clinician‐taken cervical screening and urine self‐sampling compared to 12‐week clinician‐taken cervical screening.

Participants underwent urine self‐sampling using a Colli‐pee collection device followed by clinician‐taken cervical screening samples at both 6‐ and 12‐weeks postnatal to ensure adequate sampling of shed cervical cells in the urine sample (see published protocol for full details [27]). This sample size was chosen to provide a standard error on uptake at most 2.5% on each proportion, which we judged to be suitable for assessing feasibility of a subsequent paired study design for accuracy. Eligibility criteria were people eligible for the NHS CSP (women/people with a cervix, aged over 24.5 years) recruited during pregnancy or within 6‐weeks of childbirth, irrespective of mode of birth. Potential participants were approached in both antenatal and postnatal settings or could contact the study team directly from publicised recruitment materials. Participants were recruited regardless of previous cervical screening history; participant self‐reported screening status and recollection of previous screening status was recorded. Demographic data (e.g., age group, ethnicity, sexual orientation, parity, employment, Index of Multiple Deprivation (calculated from postcode)) and factors affecting cervical cancer risk (e.g., smoking, immunosuppression and previous HPV vaccination) were collected. Cervical screening tests were performed and processed as per NHS CSP standard operating procedures. Abnormal results were managed as per NHS CSP guidelines, as were normal results of participants who were due screening. The results of those who were not due screening were managed as per the trial protocol (see [27] for full details). The anticipated recruitment period was 18 months.

At each appointment, participants completed a questionnaire about their experience and preferences after providing clinical samples (see Supporting Information—Appendix S1). Participants could select from a list of 18 words regarding the screening test, 4 positive and 14 negative, derived from descriptions of screening in previous work [17, 19, 20]. To further understand feasibility, and identify barriers to participation, participants who withdrew or declined participation were asked for their reasons via an anonymous questionnaire, with possible responses informed by previous research and PPIE feedback (see Appendices S2 and S3) [17, 19, 20].

Ethical approval for PINCS‐1 was given by the Stanmore Research Ethics Committee (IRAS project ID: 321696). The protocol was registered and a peer‐reviewed version published (ISRCTN10071810) [27]. This study was instigated following the direct request by stakeholders, when investigating methods to reduce barriers to cervical screening in the postnatal period [12]. Local Maternity Voices groups helped with design of study materials and feedback from the pre‐PINCS study participants was incorporated into study design, materials and questionnaires [19, 20].

Three NHS study sites with maternity units providing antenatal, intrapartum and postnatal care across South West England (Somerset NHS Foundation Trust, Royal Devon University Healthcare NHS Foundation Trust and Royal Cornwall Hospitals NHS Trust) recruited participants, completed study visits and data collection. Cervical samples were processed and tested in the regional cervical cytology laboratory (North Bristol NHS Trust) using the Hologic system. All urine samples were tested at the cytology laboratory in Manchester University NHS Foundation Trust using the Roche 8800 platform for urine HPV analysis [28].

Data were uploaded directly to a secure web database application, REDCap [29, 30]. The primary feasibility outcomes were attendance and recruitment rate, with 95% confidence intervals (CIs) calculated using Wilson's method. Acceptability was judged by a combination of pain scores (10‐point scale, 0 = no pain and 10 = worst pain imaginable), which were compared between timepoints using a Wilcoxon Signed Rank test, and participant reported outcomes, via descriptive percentages of Likert questionnaire responses and cytology adequacy, calculated at each time point using all available responses. Among participants with data at both 6‐ and 12‐week time points, paired analysis was performed using McNemar's test to assess changes in agreement and adequacy. We calculated the sensitivity, specificity and negative predictive values (NPV) of sampling type and time point. Using McNemar's test a single *p*‐value was calculated to assess differences in sensitivity and specificity between 2 paired tests (clinician‐taken samples at 6‐weeks, and urine self‐samples at 6‐ and 12‐weeks, versus the 12‐week clinician sample ‘gold standard’). Analyses were conducted with Stata version 18.5, R version 4.5.1.

## 3 Results

### 3.1 Primary Outcomes

#### 3.1.1 Feasibility

**FIGURE 1:**
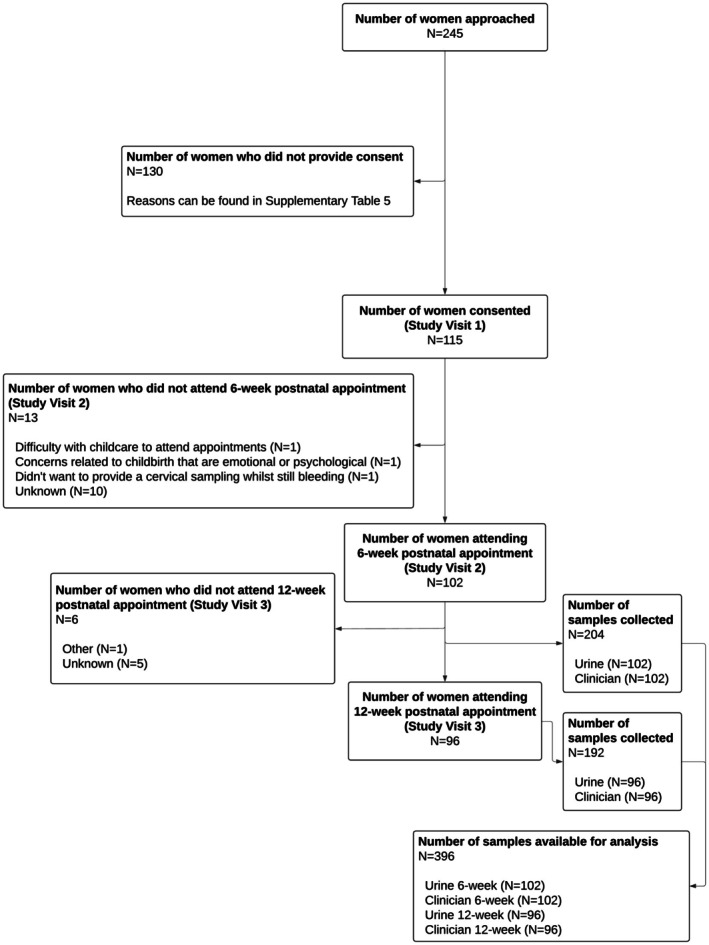
Study flow chart of participant recruitment.

Between August 2024 and March 2025, 245 pregnant or postnatal people were approached to participate in the study; 115 participants consented (47%). Of those who consented, 102 (89%, 95% CI 82%–93%) attended the 6‐week screening visit and 96 (83%, 95% CI 76%–89%) attended at both 6‐ and 12‐weeks (Figure 1). Median age was 32.3 years (Interquartile range [IQR] 29.7–34.4 years) and the median number of children was 2 (Table 1 and Table S1 for further demographic details). Of the 102 participants, 55 (53.9%) gave birth by caesarean (planned = 16 [15.7%]; emergency = 39 [38.2%]), eight (7.8%) instrumental (assisted) vaginal births, and 39 (38.2%) unassisted vaginal births (see Table S2 for further delivery and complication details). Just over half (54/102; 52.9%) of the participants attending at 6‐weeks self‐reported that they were in‐date with their cervical screening (Table S3 for further screening and HPV vaccination details).

**TABLE 1:** Participant demographic data collected via questionnaire at study visit (categories with zero participants were omitted from the table).

	Consented
	Total *N* = 115*
	Number (%)
Age category (years)	
< 30	32 (28.1%)
30–44	81 (71.1%)
45+	1 (0.9%)
Ethnicity	
White British	100 (87.7%)
Other white	6 (5.3%)
Indian	2 (1.8%)
Other Asian	1 (0.9%)
African	3 (2.6%)
Other black/African/Caribbean	2 (1.8%)
Sexual orientation	
Heterosexual	110 (96.5%)
Bisexual	4 (3.5%)
Smoking status	
Current smoker	5 (4.4%)
Current vaper	6 (5.3%)
Ex‐smoker or ex‐vaper	34 (29.8%)
Never smoked	68 (59.6%)
Highest educational attainment	
GCSE or equivalent	15 (13.2%)
A‐level or equivalent	24 (21.1%)
Undergraduate degree	48 (42.1%)
Postgraduate degree	23 (20.2%)
Other	4 (3.5%)
Employment status	
Employed	61 (53.5%)
Unemployed	4 (3.5%)
Full time parent or carer	8 (7.0%)
Parental leave	40 (35.1%)
Other	1 (0.9%)

* IMD calculated from residential postcode.

Participants who declined to participate and completed the decline questionnaire were not significantly different in age (median age 32.3; IQR 29.7–34.4) to those who chose to participate (median age 32.7; IQR 29.6–36.3) (Table S4). Other demographic data on non‐participants were limited with numbers too small for accurate comparison. The most common reasons given for non‐participation included not wanting to provide a cervical screening sample at 6‐weeks postnatal (17/80; 21.2%); travelling to the hospital for the study (13/80; 16.2%); concerns related to childbirth that were physical (8/80; 10.0%); and not enough time (7/80; 8.8%) (Table S5).

### 3.2 Secondary Outcomes

#### 3.2.1 Acceptability

##### 3.2.1.1 Experience of Clinician‐Taken Screening at 6‐ Versus 12‐Weeks

There was no difference in the median pain score (scale of 0–10) for cervical screening at different time points (median score at 6‐weeks = 1 [range 0–8]; median score at 12‐weeks = 1 [range 0–7; *p* = 0.76]) (Figure S1). There was no difference in the relative number of positive and negative words used to describe the experience of cervical screening at each time point (Figure S2; Table S6). At 6‐weeks, no participant chose the description “too soon” to describe cervical screening. Almost all participants answered every questionnaire element (94.1% for study visit 2, 94.8% for study visit 3).

**FIGURE 2:**
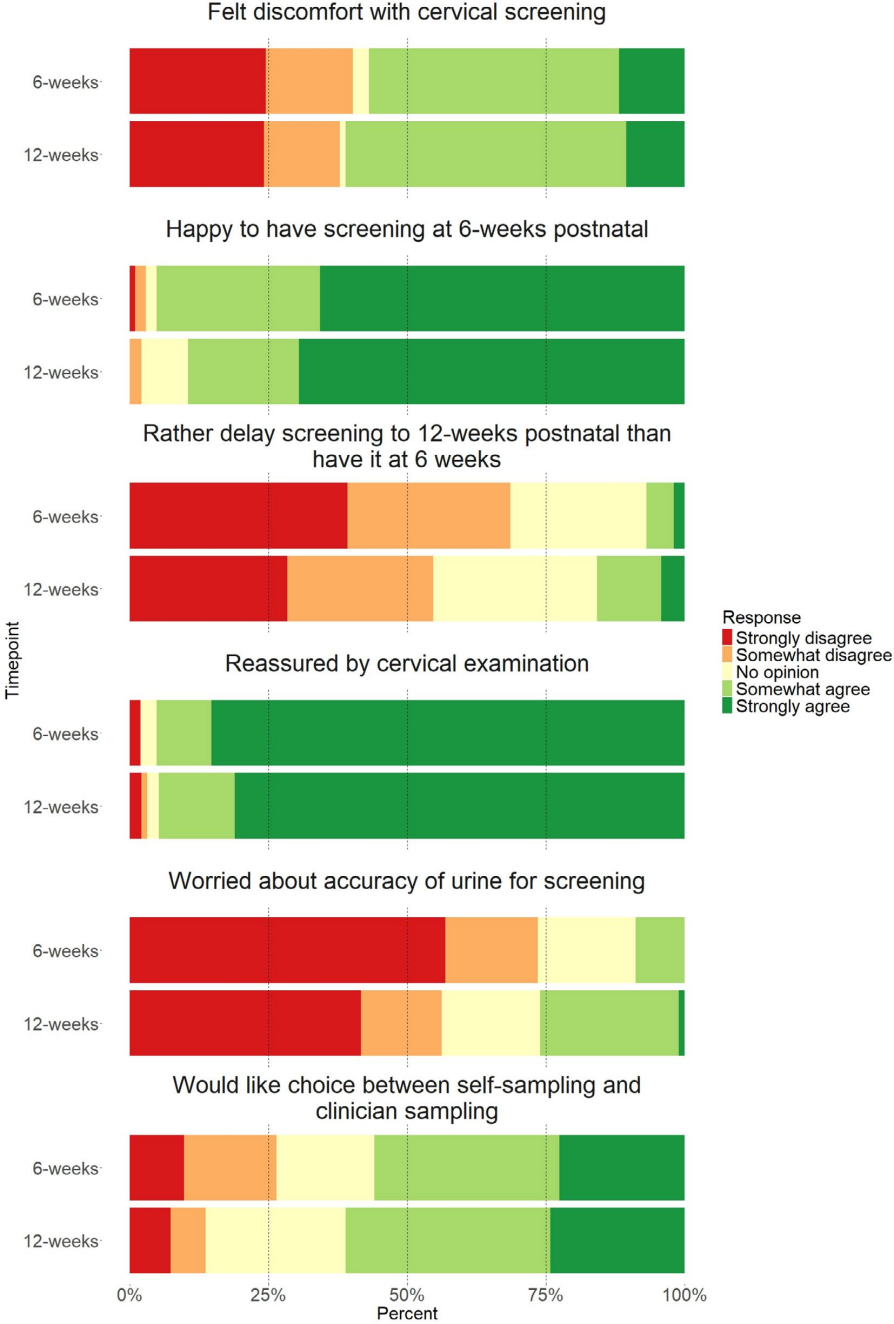
Participant experience of cervical screening at 6‐ and 12‐week sample collection from a 5‐point Likert scale (strongly disagree = red; somewhat disagree = orange; no opinion = light yellow; somewhat agree = light green; strongly agree = dark green).

At 6‐weeks postnatal, 58/102 participants (57%) agreed that they experienced discomfort whilst having clinician‐taken sampling, compared with 58/95 (61.1%) at 12 weeks postnatal (*p* = 0.44) (Figure 2). 19/102 (18.6%) agreed that they felt anxious at 6‐weeks compared with 19/95 (20.0%) at 12 weeks (*p* = 0.63) (Figure 2). However, 97/102 (95.1%) agreed that they felt reassured by the examination, compared with 90/95 (94.75%) at 12‐weeks (*p* = 1.00) (Figure 2).

When asked about future screening preferences on the visit questionnaire, most agreed that they would be happy to have cervical screening at their 6‐week postnatal check‐up: 97/102 (95%) when asked at the 6‐week visit and 85/95 (89%) when asked again at the 12‐week visit (*p* = 0.11) (Figure 2). At 6‐weeks, only 7/102 (6.9%) agreed they would have preferred waiting until 12‐weeks postnatal for their cervical screening. A few participants were worried about the accuracy of cervical screening at 6‐weeks compared with screening performed at 12‐weeks (agreed = 9/102 [8.8%] and 12/94 [12.8%] at 6‐ and 12‐weeks, respectively; *p* = 0.29). Results for other preference questions can be found in Figure S3.

#### 3.2.2 Comparison of Different Screening Timing and Methods

The inadequate rate of combination testing was 0% at 6‐ and 12‐weeks. For cytology alone, the inadequate rate was 5/101 (5.0%) at 6‐weeks and 2/96 (2.1%) at 12‐weeks (*p* = 0.18). When clinician‐taken cervical screening at 6‐weeks postnatal was compared with clinician‐taken cervical screening after 12‐weeks postnatal, the sensitivity was 80% (95% CI 44.4–97.5), with a specificity of 96.5% (95% CI 90.1 to 99.3) and a NPV of 97.6% (95% CI 91.8–99.7) (Table S7). The agreement rate of HPV test outcome between 6‐ and 12‐weeks was 94.8% (91/96).

Compared with clinician‐taken cervical screening after 12‐weeks postnatal, the sensitivity of urine self‐sampling at 6‐weeks was 60.0% (95% CI 26.2 to 87.8), with a specificity of 96.5% (95% CI 90.1–99.3) and a NPV of 95.4% (95% CI 88.6–98.7; McNemar's *p* = 0.71; NPV *p* = 0.46) (Table S8); results for urine self‐sampling at 12‐weeks were similar (See Tables S9 and S10).

Of the 11 participants referred to colposcopy for abnormal results at either or both 6‐ or 12‐weeks, one had high‐grade cervical intraepithelial neoplasia (CIN2), two had low‐grade CIN (CIN1), seven had HPV changes with no CIN, and one was normal (inflammatory changes only); all attended the offered appointment (Table S11). Importantly, the one participant with CIN2 on biopsy had positive HPV testing on both urine self‐sampling and clinician‐taken screening at both 6‐ and 12‐weeks.

##### 3.2.2.1 Adverse Events

One participant reported a urinary tract infection requiring oral antibiotics within a week of undergoing screening at 6‐weeks postnatal, although no microbiology sample was sent for confirmation.

##### 3.2.2.2 Experience of Urine Self‐Sampling for HPV

Almost all participants reported they felt confident performing the urine sample (101/102 [99.0%] agreed at 6‐weeks and 93/95 [97.9%] at 12‐weeks). Despite this, some reported feeling worried they had not performed it correctly (11/102 [10.8%] vs. 8/95 [8.4%] at 6‐ and 12‐weeks, respectively; *p* = 0.58). Interestingly, fewer were concerned about the accuracy of urine self‐sampling for cervical screening at 6‐weeks than at 12‐weeks (9/102 [8.8%] vs. 25/96 [26.0%], respectively; *p* < 0.0001). When asked whether participants would prefer a urine self‐sample to a clinician‐taken sample for cervical screening, less than half preferred urine self‐sampling (43/102 [42.2%] at 6‐weeks vs. 40/95 [42.1%] at 12‐weeks; *p* = 1.00).

##### 3.2.2.3 Preferences for Future Screening

At the end of each screening visit, participants were asked about their preference for a future screening method via the visit questionnaire (see Figure S3). The most popular answer was conventional clinician‐taken sampling (45/102 [44.1%] at 6‐weeks; 38/95 [40.0%] at 12‐weeks). A combination of methods was chosen by 29/102 (28.4%) and 24/95 (25.3%) at 6‐ and 12‐weeks, respectively, and urine sampling was chosen by 9/102 (8.8%) and 19/95 (20.0%) at the corresponding time points. Only one participant at 6‐weeks and none at 12‐weeks would choose vaginal self‐sampling, although this wasn't a modality clinically evaluated in the study. At 12‐weeks, over half agreed that they would be more likely to have screening performed at 6‐weeks postnatal, if offered as a urine self‐sample (56/95, 59.0%), with 45.1% agreeing with this statement at 6‐weeks (46/102; *p* = 0.05). Further details of participant preferences are detailed in Figure 2 and Figure S3.

## 4 Discussion

### 4.1 Main Findings

We identified that offering cervical screening at the 6‐week postnatal check‐up is acceptable to postnatal participants and that a paired‐sample, non‐inferiority study to assess the accuracy of cervical screening at 6‐weeks postnatal would be feasible. Furthermore, urine self‐sampling appeared feasible and easy to perform in a postnatal population. Given the small sample size (*n* = 102) in this feasibility study, these data suggest that urine self‐sampling might not be as accurate in postnatal individuals, although urine self‐sampling did identify the one participant with CIN2 [28, 31]. A larger, adequately powered study is required to evaluate diagnostic accuracy and comparison with vaginal self‐sampling, although the idea of vaginal self‐sampling was less acceptable to participants than either clinical‐taken cervical screening or urine self‐sampling. However, this may reflect limited familiarity with vaginal self‐sampling in this cohort, and further work will compare these different methods directly.

### 4.2 Strengths and Limitations

A key strength of this study is that the research question was generated independently by new parents and primary care staff in earlier focus groups, reflecting its relevance and importance to stakeholders and potentially contributing to higher‐than‐anticipated recruitment rates. This was achieved despite participants being required to return to hospital for study visits, rather than attending in a primary care setting. Paired‐sample testing was performed to provide head‐to‐head comparisons of both timing and methods of cervical screening, as well as in‐depth patient‐reported outcomes on their experience and preferences for different screening methods.

Importantly, this study is the first to demonstrate that screening 6 weeks after a previous screening test does not lead to a significant increase in inadequate samples on repeat testing, which has major implications for the wider screening programme outside of the postnatal period.

A possible limitation was that participants were required to attend screening appointments in secondary care, separate to their GP 6‐week postnatal check‐up. This deterred some and was a reason for non‐participation in this study, due to travel requirements involved, especially as the study was conducted in a region of rural and coastal communities, where access to secondary care is challenging. However, because participants were able to choose an appointment time in advance, and were remunerated for inconvenience, this may have paradoxically improved attendance. Despite this, 95.1% agreed or strongly agreed that they would be happy to have a clinician‐taken cervical screening test, if offered in the future as part of their GP 6‐week postnatal check‐up. This study was performed in a hospital setting, rather than primary care to limit the number of study sites for practical reasons. This may have improved the quality of the clinician‐taken samples, although the inadequate rate at 12‐weeks was 0%, comparable to that in the wider screening programme [8]. A further limitation is that this study recruited those who were both in‐date and overdue cervical screening. This was a pragmatic decision to improve uptake, although previous work found that 50% of women are out of date by the end of pregnancy and 34% (self‐reported) in our cohort were out of date for screening [12]. As this study was to estimate the number required to power a larger diagnostic test accuracy study, not a study to test the effect on screening uptake, we reasoned that adequacy rates were unlikely to relate to previous screening status.

### 4.3 Interpretation

Participants were willing to be offered cervical screening at 6‐weeks postnatal and there was no difference in pain scores compared to 12‐weeks. Most felt that this would increase the likelihood of them accepting screening, if offered alongside the 6‐week postnatal check‐up in primary care. Urine testing was less popular, and some participants were concerned about its accuracy, although most found this easy to perform. Participants were less keen on vaginal swabbing for self‐sampling, perhaps because they had no experience of this test in this study and other studies have found that some people are concerned about performing the self‐swab test correctly [32].

Although this feasibility study was not powered to define the sensitivity and specificity of cervical screening for HPV at 6‐weeks, the preliminary data suggest that the NPV was high and not significantly different to that at 12‐weeks. Urine self‐sampling appeared to be less accurate for HPV detection than anticipated in the postnatal population, compared with other studies [28]. However, the sample size was small, urine self‐sampling was positive in the one participant with CIN2 at both 6‐ and 12‐weeks, and further exploration is warranted, as is comparison with vaginal swabs, especially as these are soon to be offered within the NHS Cervical Screening Programme for non‐attenders.

## 5 Conclusion

These data warrant further exploration through a fully‐powered paired‐sample study to determine whether cervical screening at 6‐weeks postnatal is sufficiently accurate to be recommended in UK and international policies. To increase the efficiency of a future study, the diagnostic test accuracy of self‐sampling (urine and/or vaginal swabs) in a postnatal population should also be compared against conventional cervical screening at the same time.

## Author Contributions

J.M., V.A.C., A.B., K.D. and E.J.D. designed the PINCS‐1 study, with input from R.N., H.B.‐R., and L.M. V.A.C., T.D., L.J., K.E., S.L.C., J.B. and J.M. recruited participants and performed study procedures and data collection. K.H., K.D. and A.S. led testing and reporting of LBC and urine samples. V.A.C, K.C., A.B., and J.M. designed and performed the data collection and analyses. V.A.C., K.C. and J.M. drafted the manuscript with contributions from the other authors. All authors approved the final version. J.M. is guarantor for the study.

## Funding

The study was funded by a Medical Research Council Clinical Academic Research Partnership grant (J.M.—Grant Ref: MR/X030776/1) and a National Institute for Health and Care Research (NIHR) South West Peninsula Clinical Research Network Research Associate Scheme Grant (R.N.). This involved external peer review for scientific quality. The study was the idea of a patient and public focus group during a previous quality improvement study [12] and informed by feedback from an attitudes study involving 454 members of the public [27]. E.J.D. is separately supported by an NIHR Advanced Fellowship (NIHR300650). E.J.D. and L.M. are separately supported by the National Institute for Health and Care Research (NIHR) Manchester Biomedical Research Centre (NIHR203308). L.M. is separately supported by a Cancer Research UK Career Development Fellowship (RCCCDF‐May25/100006). The funding sources had no role in the design of this article but feedback from MRC peer review led to changes in study design. The views expressed are those of the authors and not necessarily those of the MRC, NIHR or the Department of Health and Social Care.

## Ethics Statement

Ethical approval was granted by the Stanmore Research Ethics Committee (IRAS project ID:321696; REC reference: 24/LO/0206), was adopted by the NIHR Clinical Research Network (CRN) Portfolio (CPMS ID 60494) and was registered on the International Standard Randomised Controlled Trial Number (ISRCTN) registry (ISRCTN10071810; https://doi.org/10.1186/ISRCTN10071810), with publication of the protocol [27].

## Consent

All patients provided written consent to take part in this study, as per the ethical approval and published protocol (above). Data were pseudonymised and no identifying information is presented.

## Conflicts of Interest

K.C. received honorarium and support from SeeGene. J.M. is a member of the NHS Cervical Screening Programme Research, Innovation and Development Committee. The other authors declare no conflicts of interest.

## Supporting information


Tables S1–S11.
Figures S1–S3.
Appendix S1–S3.

## Data Availability

Anonymised data will be made available on reasonable request to the authors, in line with ethical approval for the study. Materials are available as Supporting Information to this paper. For the purpose of open access, the author has applied a ‘Creative Commons Attribution (CC BY)’ licence to any Author Accepted Manuscript version arising from this submission. We do not consent to the contents of this paper being used to inform large language models.
