# Supplementary material for: Comparison of Cervical Screening at 6‐ and 12‐Weeks Postnatal: A Paired‐Sample Feasibility Study
Source: BJOG. 2026 Apr 1;133(10):1857–65. doi: 10.1111/1471-0528.70234 (PMC13485450; doi:10.1111/1471-0528.70234)
Supplement: Supplementary file 1 [file BJO-133-1857-s001.docx]

**Supplementary materials**

Table of Contents

[Table S1: Further demographic data	4](#_Toc223691364)

[Table S2: delivery and complication information	6](#_Toc223691365)

[Table S3: Participant cervical screening and HPV vaccination status	7](#_Toc223691366)

[Table S4: Demographic data on those approached who opted out of participation	8](#_Toc223691367)

[Table S5: Reasons provided for opting out of participation	10](#_Toc223691368)

[Table S6: The number of positive and negative words selected to describe each screening modality at 6- and 12-weeks	11](#_Toc223691369)

[Table S7: 2x2 table of 6- vs 12-week cervical screening HPV results	12](#_Toc223691370)

[Table S8: 2x2 table of 6-week urine vs. 12-week cervical screening HPV results	12](#_Toc223691371)

[Table S9: 2x2 table of 12-week urine vs. 12-week cervical screening HPV results	12](#_Toc223691372)

[Table S10: 2x2 table of 6- vs. 12-week urine self-samples HPV results	12](#_Toc223691373)

[Table S11: Timing of abnormal clinician-taken samples leading to referral to colposcopy and colposcopy outcomes	13](#_Toc223691374)

[Figure S1: Difference in median pain scores for clinician-taken cervical sample	14](#_Toc223691375)

[Figure S2: Word cloud of words used to describe clinician-taken cervical screening at 6-weeks	15](#_Toc223691376)

[Figure S3: participant preferences on a 5-point Likert scale for screening methods at both 6- and 12-week time points	16](#_Toc223691377)

[Appendix 1 – Post visit questionnaire	17](#_Toc223691378)

[17](#_Toc223691379)

[Appendix 2 - Withdrawal questionnaire	22](#_Toc223691380)

[Appendix 3 – Decline questionnaire	23](#_Toc223691381)

## Table S1: Further demographic data

|  | **Consented**  **Total N = 115***  **Number (%)** | **Attended 6-weeks**  **Total N = 102**  **Number (%)** | **Attended 12-weeks**  **Total N = 96**  **Number (%)** |
| --- | --- | --- | --- |
| **Age category (years)** |  | |  |
| <30 | 32 (28.1%) | 29 (28.4%) | 25 (26.0%) |
| 30-44 | 81 (71.1%) | 72 (70.6%) | 70 (72.9%) |
| 45+ | 1 (0.9%) | 1 (1.0%) | 1 (1.0%) |
| **Ethnicity** |  | |  |
| White British | 100 (87.7%) | 88 (86.3%) | 85 (88.5%) |
| Other white | 6 (5.3%) | 6 (5.9%) | 6 (6.2%) |
| Indian | 2 (1.8%) | 2 (2.0%) | 1 (1.0%) |
| Other Asian | 1 (0.9%) | 1 (1.0%) | 1 (1.0%) |
| African | 3 (2.6%) | 3 (2.9%) | 2 (2.1%) |
| Other black/African/Caribbean | 2 (1.8%) | 2 (2.0%) | 1 (1.0%) |
| **Sexual orientation** |  | |  |
| Heterosexual | 110 (96.5%) | 98 (96.1%) | 92 (95.8%) |
| Bisexual | 4 (3.5%) | 4 (3.9%) | 4 (4.2%) |
| **Smoking status** |  |  |  |
| Current smoker | 5 (4.4%) | 4 (3.9%) | 4 (4.2%) |
| Current vaper | 6 (5.3%) | 4 (3.9%) | 4 (4.2%) |
| Ex-smoker or ex-vaper | 34 (29.8%) | 30 (29.4%) | 28 (29.2%) |
| Never smoked | 68 (59.6%) | 64 (62.7%) | 60 (62.5%) |
| **HIV status** |  | |  |
| Positive | 0 (0.0%) | 0 (0.0%) | 0 (0.0%) |
| Negative | 112 (98.2%) | 100 (98.0%) | 94 (97.9%) |
| Unknown | 2 (1.8%) | 2 (2.0%) | 2 (2.1%) |
| **Immunosuppressant use** |  | |  |
| Yes | 0 (0.0%) | 0 (0.0%) | 0 (0.0%) |
| No | 112 (98.2%) | 100 (98.0%) | 94 (97.9%) |
| Unknown | 2 (1.8%) | 2 (2.0%) | 2 (2.1%) |
| **Marital status** |  | |  |
| Single | 11 (9.6%) | 10 (9.8%) | 9 (9.4%) |
| Married or domestic partnership | 99 (86.8%) | 90 (88.2%) | 85 (88.5%) |
| Other | 2 (1.8%) | 2 (2.0%) | 2 (2.1%) |
| **Highest educational attainment** |  | |  |
| GCSE or equivalent | 15 (13.2%) | 11 (10.8%) | 11 (11.5%) |
| A-level or equivalent | 24 (21.1%) | 21 (20.6%) | 19 (19.8%) |
| Undergraduate degree | 48 (42.1%) | 43 (42.2%) | 41 (42.7%) |
| Postgraduate degree | 23 (20.2%) | 23 (22.5%) | 22 (22.9%) |
| Other | 4 (3.5%) | 4 (3.9%) | 3 (3.1%) |
| **Employment status** |  | |  |
| Employed | 61 (53.5%) | 55 (53.9%) | 53 (55.2%) |
| Unemployed | 4 (3.5%) | 3 (2.9%) | 3 (3.1%) |
| Full time parent or carer | 8 (7.0%) | 7 (6.9%) | 6 (6.2%) |
| Parental leave | 40 (35.1%) | 36 (35.3%) | 33 (34.4%) |
| Other | 1 (0.9%) | 1 (1.0%) | 1 (1.0%) |

|  | **Consented**  **Total N = 115***  **Number (%)** | **Attended 6-weeks**  **Total N = 102**  **Number (%)** | **Attended 12-weeks**  **Total N = 96**  **Number (%)** |
| --- | --- | --- | --- |
| **Index of Multiple Deprivation (IMD)*** |  | |  |
| Quintile 1 (most deprived, deciles 1-2) | 8 (7.0%) | 6 (5.9%) | 5 (5.2%) |
| Quintile 2 (deciles 3-4) | 21 (18.4%) | 20 (17.9%) | 20 (20.8%) |
| Quintile 3 (deciles 5-6) | 38 (33.3%) | 33 (32.4%) | 30 (31.3%) |
| Quintile 4 (deciles 7-8) | 22 (19.3%) | 21 (20.6%) | 20 (20.8%) |
| Quintile 5 (least deprived, deciles 9-10) | 13 (11.4%) | 10 (9.8%) | 9 (9.4%) |
| Unknown | 12 (10.5%) | 12 (11.8%) | 12 (12.5%) |
| **Number of children responsible for** |  | |  |
| 1 | 50 (43.9%) | 46 (45.1%) | 42 (43.8%) |
| 2 | 43 (37.7%) | 41 (40.2%) | 39 (40.6%) |
| 3 | 13 (11.4%) | 8 (7.8%) | 8 (8.3%) |
| 4 | 6 (5.3%) | 5 (4.9%) | 5 (5.2%) |
| 5+ | 1 (0.9%) | 1 (1.0%) | 1 (1.0%) |
| Prefer not to say | 1 (0.9%) | 1 (1.0%) | 1 (1.0%) |

*115 women consented but only 114 filled out the maternal details questionnaire.

## Table S2: delivery and complication information

|  | **Attended 6-weeks**  **Total N = 102**  **Number (%)** | **Attended 12-weeks**  **Total N = 96**  **Number (%)** |
| --- | --- | --- |
| **Mode of delivery in most recent pregnancy** | | |
| Unassisted vaginal birth | 39 (38.2%) | 38 (39.6%) |
| Instrumental (assisted) vaginal birth | 8 (7.8%) | 7 (7.3%) |
| Planned caesarean birth | 16 (15.7%) | 16 (16.7%) |
| Emergency caesarean birth | 39 (38.2%) | 35 (36.5%) |
| **Perineal tear*** | | |
| 1^st^ degree | 5 (4.9%) | 5 (5.2%) |
| 2^nd^ degree | 20 (19.6%) | 19 (19.8%) |
| 3^rd^ degree | 5 (4.9%) | 5 (5.2%) |
| No tear | 14 (13.7%) | 13 (13.5%) |
| Episiotomy (*may also have other degree of tear) | 10 (9.8%) | 9 (9.4%) |
| N/A (caesarean birth) | 51 (50%) | 48 (50%) |
| **Delivery complications*** | | |
| Heavy bleeding at delivery | 12 (11.8%) | 11 (11.5%) |
| Heavy bleeding after delivery | 4 (3.9%) | 4 (4.2%) |
| Infection | 9 (8.8%) | 9 (9.4%) |
| Retained placenta | 3 (2.9%) | 3 (3.1%) |
| Prolonged hospital admission | 15 (14.7%) | 13 (13.5%) |
| Baby admitted to neonatal unit | 6 (5.9%) | 6 (6.2%) |
| Other | 14 (13.7%) | 12 (12.5%) |
| **Postnatal complications*** | | |
| Re-admission to hospital | 8 (7.8%) | 7 (7.3%) |
| Impaired perineal healing | 1 (1%) | 1 (1%) |
| Perineal pain | 3 (2.9%) | 3 (3.1%) |
| Caesarean wound problems | 9 (8.8%) | 8 (8.3%) |
| Infection | 7 (6.9%) | 6 (6.2%) |
| Postnatal depression | 1 (1%) | 1 (1%) |
| Feeding complications | 10 (9.8%) | 9 (9.4%) |
| None | 63 (61.8%) | 59 (61.5%) |
| Other | 11 (10.8%) | 9 (9.4%) |

*may have more than 1 complication in each category so % may add up to >100% in each section

## Table S3: Participant cervical screening and HPV vaccination status

|  | **Consented**  **Total N = 115***  **Number (%)** | **Attended 6-weeks**  **Total N = 102**  **Number (%)** | **Attended 12-weeks**  **Total N = 96**  **Number (%)** |
| --- | --- | --- | --- |
| **Cervical screening status** | | |  |
| Up-to-date | 59 (51.8%) | 54 (52.9%) | 52 (54.2%) |
| Not up-to-date | 41 (36.0%) | 35 (34.3%) | 31 (32.3%) |
| Don’t know | 8 (7.0%) | 8 (7.8%) | 8 (8.3%) |
| 1^st^ invite received in this pregnancy | 6 (5.3%) | 5 (4.9%) | 5 (5.2%) |
| **Previous abnormal screening test** | | |  |
| HPV positive | 12 (10.5%) | 11 (10.8%) | 11 (11.5%) |
| Abnormal cytology | 14 (12.3%) | 13 (12.7%) | 13 (13.5%) |
| No | 89 (78.1%) | 79 (77.5%) | 73 (76.0%) |
| Don’t know | 1 (0.9%) | 1 (1.0%) | 1 (1.0%) |
| **Previous treatment/investigation of cervix** | | |  |
| Biopsy | 7 (6.1%) | 7 (6.9%) | 7 (7.3%) |
| Excision | 6 (5.3%) | 4 (3.9%) | 4 (4.2%) |
| Ablation | 2 (1.8%) | 2 (2%) | 2 (2.1%) |
| No | 96 (84.2%) | 87 (85.3%) | 81 (84.4%) |
| Don’t know | 3 (2.6%) | 3 (2.9%) | 3 (3.1%) |
| Other | 1 (0.9%) | 0 (0.0%) | 0 (0.0%) |
| **HPV vaccination status** | | |  |
| Vaccinated through vaccination programme | 48 (42.1%) | 44 (43.1%) | 43 (44.8%) |
| Vaccinated privately | 1 (0.9%) | 1 (1%) | 1 (1%) |
| No | 47 (41.2%) | 43 (42.2%) | 40 (41.7%) |
| Don’t know | 18 (15.8%) | 14 (13.7%) | 12 (12.5%) |

*115 women consented but only 114 filled out the maternal details questionnaire.

## Table S4: Demographic data on those approached who opted out of participation

|  | **Total Opted out**  **N = 22**  **Number (%)** | **Total consented**  **N = 115***  **Number (%)** |
| --- | --- | --- |
| **Employment status** |  | **p = 0.01** |
| Employed | 12 (54.6%) | 61 (53.5%) |
| Unemployed | 0 (0.0%) | 4 (3.5%) |
| Full-time parent or carer | 7 (31.8%) | 8 (7.0%) |
| Parental leave | 3 (13.6%) | 40 (35.1%) |
| Other | 0 (0.0%) | 1 (0.9%) |
| **Highest education level** |  | **p = 0.006** |
| GCSE/ O-level or equivalent | 7 (36.8%) | 15 (13.2%) |
| A- Level or equivalent | 4 (21.1%) | 24 (21.1%) |
| Undergraduate degree | 2 (10.5%) | 48 (42.1%) |
| Postgraduate degree | 4 (21.1%) | 23 (20.2%) |
| None | 1 (5.3%) | 0 (0.0%) |
| Other | 1 (5.3%) | 4 (3.5%) |
| **Ethnicity** |  | **p = 0.90** |
| White British | 22 (100%) | 100 (87.7%) |
| Other white | 0 (0.0%) | 6 (5.3%) |
| Indian | 0 (0.0%) | 2 (1.8%) |
| Other Asian | 0 (0.0%) | 1 (0.9%) |
| African | 0 (0.0%) | 3 (2.6%) |
| Other black/African/Caribbean | 0 (0.0%) | 2 (1.8%) |
| **Gender identity** |  | **NA** |
| Women | 22 (100%) | 114 (100.0%) |
| **Sexual Orientation** |  | **p = 0.03** |
| Heterosexual or straight | 18 (90%) | 110 (96.5%) |
| Lesbian or gay | 2 (10%) | 0 (0.0%) |
| Bisexual | 0 (0.0%) | 4 (3.5%) |
| **Up-to-date with cervical screening** |  | **p = 0.31** |
| Yes | 14 (70%) | 59 (51.8%) |
| No | 4 (20%) | 41 (36.0%) |
| Unsure | 2 (10%) | 8 (7.0%) |
| 1st invite received in this pregnancy | 0 (0.0%) | 6 (5.3%) |

|  | **Total Opted out**  **N = 22**  **Number (%)** | **Total consented**  **N = 115***  **Number (%)** |
| --- | --- | --- |
| **Pregnant or postnatal** |  | **p = 0.014** |
| Postnatal | 22 (100%) | 90 (78.9%) |
| Pregnant | 0 (0.0%) | 24 (21.1%) |
| **Likelihood of the participating if in local GP than hospital**** |  | **NA** |
| Very likely | 6 (27.3%) | - |
| Likely | 5 (22.7%) | - |
| Neither likely or unlikely | 4 (18.2%) | - |
| Unlikely | 1 (4.6%) | - |
| Very unlikely | 6 (27.3%) | - |
| Total | 22 (100%) | - |

*115 participants consented but only 114 filled out the maternal details questionnaire.

** Only asked in initial opt out questionnaire

## Table S5: Reasons provided for opting out of participation

| **Reasons for declining*** | **Number (%)**  **total N = 80**** |
| --- | --- |
| Not enough time | 7 (8.8%) |
| Don't take part in research | 2 (2.5%) |
| Didn't feel that I understood the study | 0 (0.0%) |
| Didn't want to provide a cervical sample at all | 5 (6.2%) |
| Didn't want to provide two cervical samples | 3 (3.8%) |
| Didn't want to provide a cervical sample 6 weeks after delivery | 17 (21.2%) |
| Didn't want to provide a urine sample | 0 (0.0%) |
| Didn't want to provide two urine samples | 0 (0.0%) |
| Didn't want to travel to the hospital for the study | 13 (16.2%) |
| Difficulty with childcare to attend appointments | 6 (7.5%) |
| Concerns related to childbirth that are physical | 8 (10%) |
| Concerns related to childbirth that are emotional or psychological | 1 (1.2%) |

* Multiple responses were permitted. Percentages reflect the proportion of respondents selecting each option, not the proportion of total responses.

**50 potential participants did not respond to opt out questionnaire.

## Table S6: The number of positive and negative words selected to describe each screening modality at 6- and 12-weeks

|  | **Number (%)** | | | |
| --- | --- | --- | --- | --- |
|  | **6-weeks urine** | **12-weeks urine** | **6-weeks clinician-taken cervical screening** | **12-weeks clinician-taken cervical screening** |
| **Total** | 102 (100.0) | 96 (100.0) | 102 (100.0) | 95 (100.0) |
| **Number of positive words selected** |  |  |  |  |
| 0 | 5 (4.9) | 1 (1.0) | 28 (27.4) | 15 (15.8) |
| 1 | 70 (68.6) | 68 (70.8) | 41 (40.2) | 41 (43.2) |
| 2 | 14 (13.7) | 12 (12.5) | 17 (16.7) | 17 (17.9) |
| 3 | 12 (11.8) | 7 (7.3) | 13 (12.8) | 15 (15.8) |
| 4 | 1 (1.0) | 8 (8.3) | 3 (2.9) | 7 (7.4) |
| **Number of negative words selected** |  |  |  |  |
| 0 | 101 (99.0) | 95 (99.0) | 47 (46.1) | 53 (55.8) |
| 1 | 1 (1.0) | 1 (1.0) | 32 (31.4) | 23 (24.2) |
| 2 | 0 (0.0) | 0 (0.0) | 11 (10.8) | 8 (8.4) |
| 3 | 0 (0.0) | 0 (0.0) | 7 (6.9) | 5 (5.3) |
| 4 | 0 (0.0) | 0 (0.0) | 3 (2.9) | 2 (2.1) |
| 5 | 0 (0.0) | 0 (0.0) | 1 (1.0) | 2 (2.1) |
| 6 | 0 (0.0) | 0 (0.0) | 1 (1.0) | 1 (1.1) |
| 7 | 0 (0.0) | 0 (0.0) | 0 (0.0) | 1 (1.1) |

## Table S7: 2x2 table of 6- vs 12-week cervical screening HPV results

|  | 12-weeks HPV+ | 12-weeks HPV- |
| --- | --- | --- |
| 6-weeks HPV+ | 8 | 3 |
| 6-weeks HPV- | 2 | 83 |

HPV negative (HPV-); HPV positive (HPV+)

## Table S8: 2x2 table of 6-week urine vs. 12-week cervical screening HPV results

|  | 12-weeks clinician HPV+ | 12-weeks clinician HPV- |
| --- | --- | --- |
| 6-weeks urine HPV+ | 6 | 3 |
| 6-weeks urine HPV- | 4 | 83 |

HPV negative (HPV-); HPV positive (HPV+)

## Table S9: 2x2 table of 12-week urine vs. 12-week cervical screening HPV results

|  | 12-weeks clinician HPV+ | 12-weeks clinician HPV- |
| --- | --- | --- |
| 12-weeks urine HPV+ | 5 | 2 |
| 12-weeks urine HPV- | 5 | 83 |

HPV negative (HPV-); HPV positive (HPV+)

## Table S10: 2x2 table of 6- vs. 12-week urine self-samples HPV results

|  | 12-weeks HPV+ | 12-weeks HPV- |
| --- | --- | --- |
| 6-weeks HPV+ | 6 | 3 |
| 6-weeks HPV- | 1 | 85 |

HPV negative (HPV-); HPV positive (HPV+)

## Table S11: Timing of abnormal clinician-taken samples leading to referral to colposcopy and colposcopy outcomes

| **Study ID** | **Timing of abnormal referral sample** | **Clinician-taken cervical screening sample** | | | | **Urine self-sample** | | | **Colposcopic impression** | **Final diagnosis** |
| --- | --- | --- | --- | --- | --- | --- | --- | --- | --- | --- |
|  |  | **6-week HPV** | **6-week cytology** | **12-week HPV** | **12-week cytology** | **6-week urine HPV** | | **12-week urine HPV** |  |  |
| 318-17 | Both study samples | Negative | Borderline | Negative | Borderline | Negative | Negative | | Metaplasia | HPV |
| 318-23 | 6-week sample | Positive | Low-grade dysk | Negative | Negative | Positive | Negative | | Low grade | HPV |
| 318-27 | Both study samples | Negative | Borderline | Negative | Borderline | Negative | Negative | | HPV | HPV |
| 318-35 | 12-week sample | Negative | Negative | Negative | Borderline | Negative | Positive | | HPV | CIN1 |
| 318-57 | Both study samples | Positive | Low-grade dysk | Positive | Low grade dysk | Negative | Negative | | Low grade | HPV |
| 318-60 | Both study samples | Positive | Low-grade dysk | Positive | Low grade dysk | Negative | Negative | | Low grade | HPV |
| 318-62 | Both study samples | Negative | Borderline | Negative | High grade dysk | Negative | Negative | | HPV | HPV |
| 318-75 | Both study samples | Negative | Low grade dysk | Negative | Low grade dysk | Negative | Negative | | Normal | Normal |
| 318-126 | Both study samples | Positive | Low grade dysk | Positive | Low grade dysk | Positive | Positive | | Low grade | CIN2 |
| 318-175 | Both study samples | Positive | Low grade dysk | Positive | Low grade dysk | Positive | Negative | | Invasion | HPV |
| 375-1 | Both study samples | Negative | Borderline | Negative | Borderline | Negative | Negative | | Low grade | CIN1 |

dys = dyskaryosis; HPV = human papillomavirus

## Figure S1: Difference in median pain scores for clinician-taken cervical sample

10-point scale (0 no pain; 10 maximum pain). Bold line = median pain score; box: interquartile range (IQR); error bars = 95% confidence interval.

## Figure S2: Word cloud of words used to describe clinician-taken cervical screening at 6-weeks

## Figure S3: participant preferences on a 5-point Likert scale for screening methods at both 6- and 12-week time points

## Appendix 1 – Post visit questionnaire

## Appendix 2 - Withdrawal questionnaire

## Appendix 3 – Decline questionnaire
